# Preparation of Metal Nitride Particles Using Arc Discharge in Liquid Nitrogen

**DOI:** 10.3390/nano11092214

**Published:** 2021-08-28

**Authors:** Yoon Sik Park, Satoshi Kodama, Hidetoshi Sekiguchi

**Affiliations:** Department of Chemical Science and Engineering, School of Materials and Chemical Technology, Tokyo Institute of Technology, Meguro-ku, Tokyo 152-8550, Japan; skodama@chemeng.titech.ac.jp (S.K.); hsekiguc@chemeng.titech.ac.jp (H.S.)

**Keywords:** titanium nitride, aluminum nitride, in-liquid plasma, liquid nitrogen, submerged arc discharge

## Abstract

A simple process to synthesize metal nitride particles was proposed using submerged arc discharge plasma in liquid nitrogen. Gibbs standard free energy was considered for the selection of the nitride-forming materials. In this study, titanium (Ti) and aluminum (Al) electrodes were used as raw materials for nitride particle preparation. Liquid nitrogen acted as a dielectric medium as well as a nitridation source in this process. A copper electrode was also used as a non-reactive material for comparison with the reactive Ti and Al electrodes. As the operating conditions of the experiments, the arc discharge current was varied from 5 A (low-power mode) to 30 A (high-power mode). The formation of titanium nitride (TiN) and aluminum nitride (AlN) was confirmed in the particles prepared in all experimental conditions by X-ray powder diffraction (XRD). The observation using a field emission scanning electron microscope (FE-SEM) and a field emission transmission electron microscope (FE-TEM) indicated that the synthesized TiN particles showed a cubic morphology, whereas AlN particles containing unreacted Al showed a spherical morphology. The experiments using different metal electrode configurations showed that the anode generated most of the particles in this process. Based on the obtained results, a particle formation mechanism was proposed.

## 1. Introduction

Metal nitrides are gaining popularity due to their physicochemical stability advantages, such as high thermal strength and excellent hardness [1,2,3]. Metal nitrides have been prepared using a variety of methods [1,2,3,4,5,6,7,8]. Direct nitridation and vapor-phase nitridation are the most commonly used methods. The former produces metal nitrides by heating metal powder at high temperatures with nitrogen or ammonia gas. At high temperatures, the latter uses vaporized precursors such as metal chlorides, to begin with, and reactive gases such as ammonia. Fine particles are commonly obtained through vapor-phase nitridation, according to popular belief. However, there are several limitations to the vapor-phase reaction. First, the starting material determines the purity of the final products. Moreover, when corrosive gases such as chloride and ammonia are used, anti-corrosion materials must be used for reactor materials. In addition, it is difficult to handle in the atmosphere in the case of using volatile compounds. Lastly, non-oxide-based particles need to be stored in an inert atmosphere.

Various plasma methods are currently being investigated for the synthesis of nitride particles. Thermal plasma processing [9,10,11,12] and plasma spray [13] have been reported for the preparation of Ti and Al nitrides. However, products contain oxide species due to imperfect nitridation and still use corrosive ammonia gas as a nitrogen source.

The in-liquid discharge has received much attention due to its simple operation process and novel liquid–plasma interaction properties that differ from conventional chemical solution methods [14,15]. It also has the advantage of being a low-cost process because expensive equipment such as special vacuum chambers and gas lining systems with anti-corrosion materials are not required for solution plasma. In many studies, water has been used as a liquid medium due to its unique properties and ease of use [16,17,18]. Some researchers have adopted precursors soluble in liquid to obtain metal or metal oxide nanoparticles [19,20,21,22,23,24,25]. It has been reported that liquid–plasma interaction produces reducing agents, enabling faster and unique reduction of metal ions [26]. Other researchers have reported particle synthesis using electrodes as a raw material in liquid discharge [16,27,28,29,30,31]. In particular, the use of arc discharge, in which currents of several amperes flow, has been widely reported in this field [17,32,33]. It is caused by the high temperature generated in an arc discharge, which can erode metal electrodes. Previous studies have demonstrated the synthesis of metal nanoparticles and oxide nanoparticles using in-liquid arc discharge. The use of electrolytes has been studied because it aids in generating stable arc discharge in a liquid environment [16,17,25,26]. Although electrolytes play an important role in determining the particle-phase composition or morphology, adding electrolytes complicates the reaction by introducing many electrochemical side reactions [32,33,34,35].

Recently, there have been studies on obtaining particles from the electrodes directly without such complex chemical reactions using plasma in liquid nitrogen. Satsuta et al. investigated the effect of discharge behavior by changing the electrode material. As a product, they also obtained Al, Ti, V, Fe, Ni, Cu, Zr, Nb, Mo, Ta, and W micro-size particles using a DC power supply [36]. Belmonte et al. also reported synthesizing Cu, Ag, Pb, CoNi, and ZnO nanoparticles using a nano-pulse power supply [30,37,38,39,40,41,42]. Sano et al. presented the synthesis of Pt-supported carbon nanohorns for fuel cell electrodes [43]. Moreover, some studies have been reported to synthesize carbon-based functional materials [44]. Most of these studies focused on plasma behaviors and their effects on the electrodes and products.

In this study, as a novel and simple process for metal nitride particle preparation, submerged arc discharge plasma in liquid nitrogen was used, where liquid nitrogen acted as a nitrogen source as well as a dielectric medium. The process is environmentally friendly since corrosive gases or chemical additives such as electrolytes and reducing agents are not used and wastewater is not produced during the whole process. In previous studies, Fink et al. reported that the breakdown voltage of liquid nitrogen under atmospheric pressure is approximately 50 kV/mm [45,46]. Thus, a high-power supply was applied for liquid nitrogen discharge here. Nitride fine particles were synthesized from the electrode directly by submerged arc discharge in liquid nitrogen. The operation process was simplified because particle preparation was performed using one-pot synthesis and in the absence of any additional additives. Considering these points, the particle formation mechanism was discussed.

## 2. Experimental Section

### 2.1. Selection of Metal Electrodes

**Figure 1:**
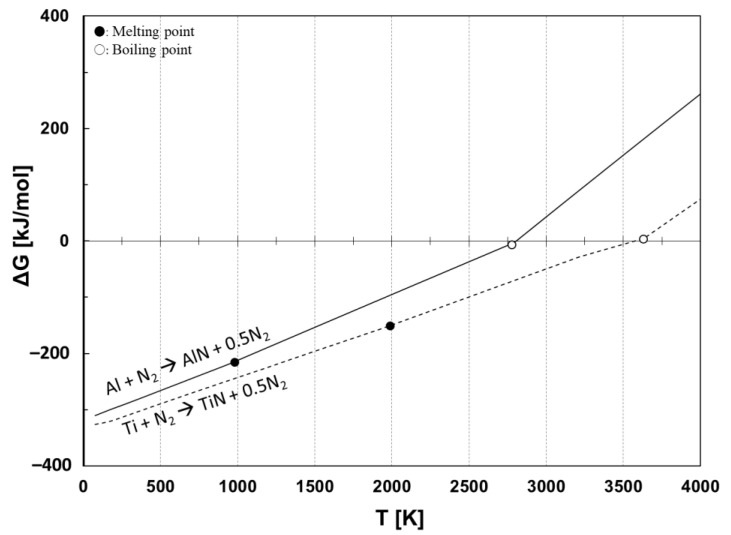
Gibbs standard free energy for synthesizing nitrides from Ti and Al electrodes.

Before selecting the electrode material, thermodynamic equilibrium calculation was considered for nitride-forming materials. Gibbs standard free energy was calculated using the chemical thermodynamics computing program (FactSage, Thermfact/CRCT, Montreal, Quebec, Canada, and GTT-Technologies, Herzogenrath, Aachen, Germany). Figure 1 shows the Gibbs standard free energy (ΔG°) change as a function of temperature for synthesizing titanium nitride (TiN) and aluminum nitride (AlN) from their metals, titanium (Ti) and aluminum (Al), respectively. The marked circles and blank circles indicate, respectively, the melting point and the boiling point of the metals. According to Figure 1, the nitridation of Ti and Al materials shows a negative ΔG° until around their boiling point temperatures, indicating that Ti and Al react with nitrogen and synthesize nitrides. Based on the consideration, Ti and Al were chosen as starting materials for the electrodes. A copper (Cu) electrode was also used as a non-reactive material compared to the reactive Ti and Al electrodes.

### 2.2. Preparation of Metal Nitride Particles

**Figure 2:**
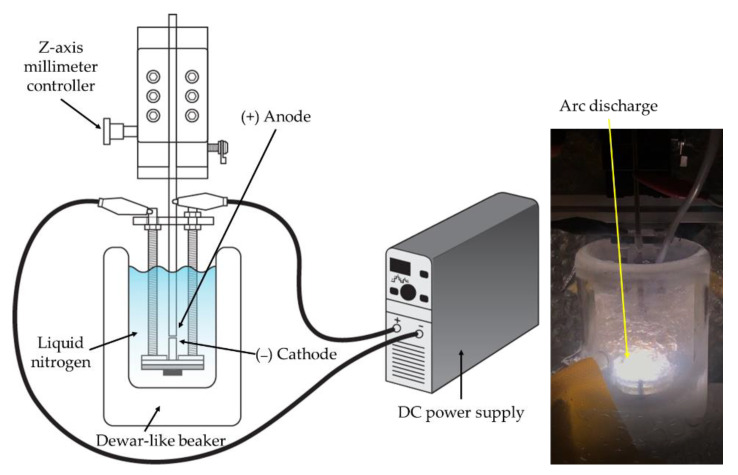
Schematic of experimental setup (**left**) and actual picture of arc discharge in liquid nitrogen (**right**).

A submerged arc discharge system was developed for the preparation of the metal nitride particles. A schematic of the direct current (DC) arc discharge system and the actual picture of arc discharge in liquid nitrogen are shown in Figure 2. A Dewar flask was used for the liquid nitrogen container to minimize the heat transfer to the surroundings. A diamond blade cut the electrodes to adjust their length. To minimize impurities such as an oxidized layer and organics stains, the electrode surface was ground by sandpaper, followed by ethanol washing. Electrodes were arranged vertically to manipulate the gap distance more feasibly during the discharge. The electrode position was precisely controlled by an anode holder equipped with a *z*-axis millimeter controller. An arc discharge was generated between two submerged metal electrode rods in liquid nitrogen. The experiments used two kinds of electrode configurations: identical metal electrode configuration and different metal electrode combinations. In the identical metal electrode configuration, Ti (99.5%, Nilaco, Tokyo, Japan), Al (99.9%, Nilaco, Tokyo, Japan), and Cu (99.9%, Nilaco, Tokyo, Japan) electrodes (∅ 3 mm) were used for both electrodes to prepare particles. In different metal electrode combinations, Ti and Cu electrodes were used for the cathode and anode, respectively, or vice versa to investigate the role of the cathode and the anode in the arc discharge for the particle preparation process.

A DC power supply (YE-200BL3, Panasonic, Osaka, Japan) was applied to generate in-liquid arc discharge. The current was set as a process parameter of the experiment. At the identical metal electrode configuration, currents of 5 A (low-power mode) and 30 A (high-power mode) were conducted. In contrast, the experiments with different metal electrode combinations were carried out only at a current of 30 A. The detailed operating conditions are summarized in Table 1, where Ti–Cu refers to Ti as the anode and Cu as the cathode, whereas Cu–Ti indicates the opposite combination.

The produced Ti particle samples at currents of 5 and 30 A were named Ti 5 A and Ti 30 A, respectively, in this paper. The same abbreviation was also applied to Al and Cu particle samples. Ti–Cu and Cu–Ti samples were also labeled Ti–Cu 30 A and Cu–Ti 30 A, respectively.

To ensure clear vision inside the vessel, dry nitrogen gas was supplied before the start of the experiments to suppress ice formation on the container’s outer surface. To start the discharge, the electrodes were placed in direct contact with no gap between them. The current was then applied while the electrodes were carefully separated. When the electrodes were detaching, a breakdown discharge was produced. Following the discharge, the electrode gap distance was controlled. The arc discharge lasted several minutes in Ti, approximately a minute in Cu, and only a few seconds in Al. This differed depending on the electrode materials and current conditions.

Because liquid nitrogen evaporated and electrodes were consumed during the experiments, a proper amount of liquid nitrogen was supplied. After the experiments, whole parts were dried to let liquid nitrogen vaporize. The collected samples were classified into two types, namely coarse particles and fine particles. Coarse particles were collected from the reactor wall and bottom using a spatula. Fine particles that remained in the reactor were collected using ethanol (99.5%, Kanto Chemical, Tokyo, Japan) washing with sonication.

### 2.3. Characterization

The crystalline phases of the coarse particle samples were investigated by X-ray diffraction (XRD, Mini Flex 600, Rigaku, Tokyo, Japan). The obtained XRD peaks were assigned based on the database of PDXL software (Rigaku, Tokyo, Japan).

The surface morphologies of the obtained particles and electrodes were observed with a scanning electron microscope (SEM, VE-9800, Keyence, Osaka, Japan) for low magnification and with a field emission scanning electron microscope (FE-SEM, SU9000, Hitachi, Tokyo, Japan, and JSM-7500F, Jeol Ltd., Tokyo, Japan) for high magnification. In operation with SU9000, the samples of Al were coated with osmium (Os) with approximately 5 nm thickness using Os plasma coating (Neoc-pro, Meiwafosis, Tokyo, Japan) before observing the samples. In the case of Ti particles, the existence of the nitrogen species was examined using energy-dispersive spectroscopy (EDS, Ametek, Genesis-APEX, Berwyn, PA, USA).

The morphologies of the synthesized nanoparticles were also observed by a field emission transmission electron microscope (FE-TEM, JEM-2010F, Jeol Ltd., Japan). The crystalline particles were identified by a selected-area electron diffraction (SAED) pattern.

## 3. Results

### 3.1. Morphologies of the Prepared Particles in Liquid Nitrogen Discharge

#### 3.1.1. FE-SEM Observation

**Figure 3:**
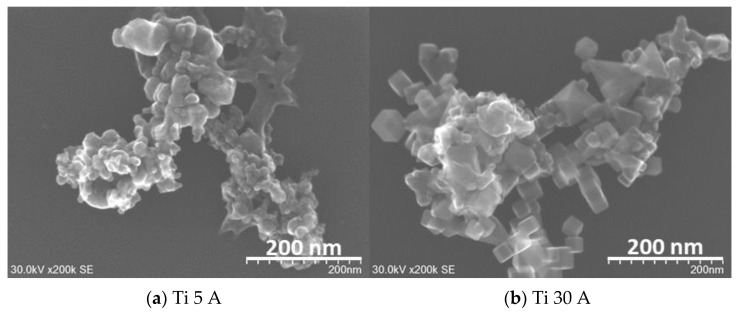

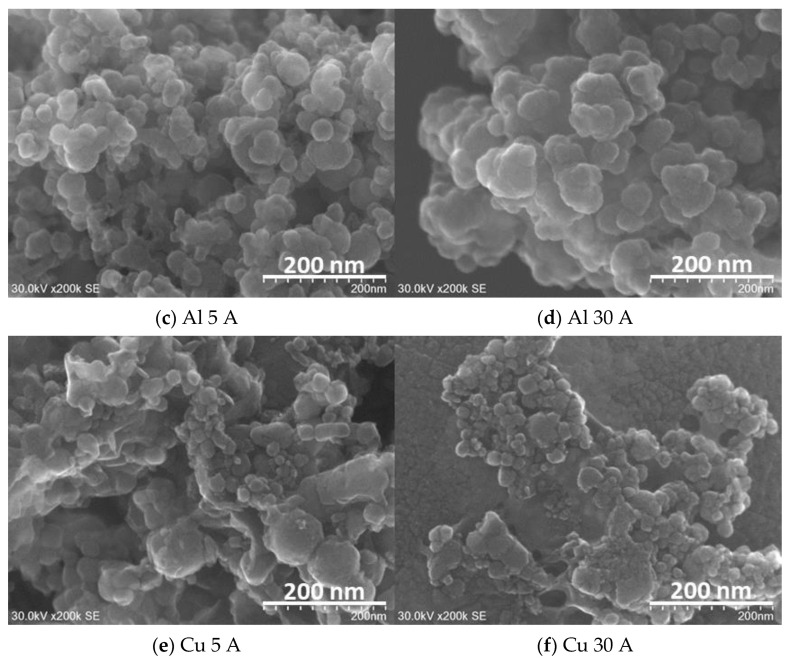
FE-SEM (SU9000) images of the prepared nanoparticles using (**a**,**b**) Ti, (**c**,**d**) Al, and (**e**,**f**) Cu electrodes at a current of 5 and 30 A. Detailed descriptions are placed below in each panel.

**Figure 4:**
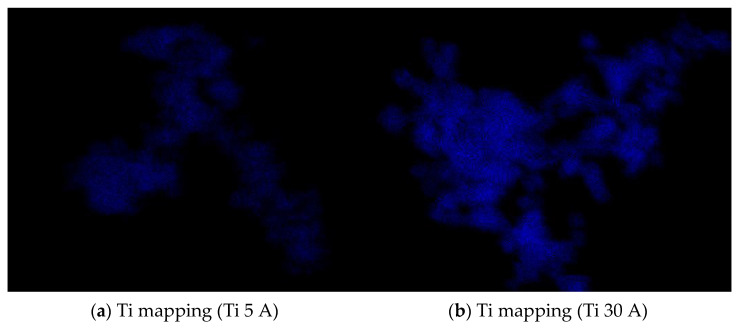

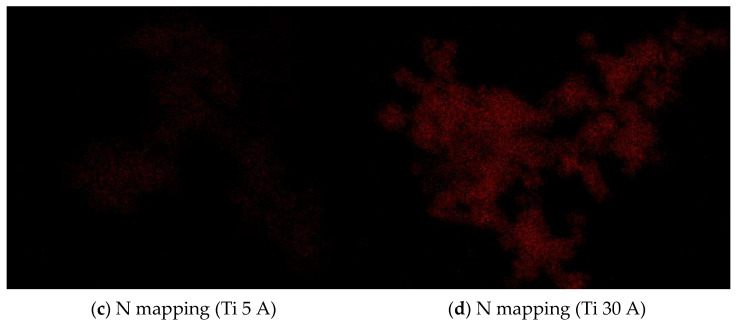
FE-SEM pictures of Ti 5 A and Ti 30 A and corresponding EDS (**a**,**b**) Ti elemental and (**c**,**d**) N elemental mapping images. Detailed descriptions are placed below each panel.

The FE-SEM (SU9000) images of prepared fine particles using Ti, Al, and Cu electrodes are shown in Figure 3. Figure 3a,b shows the Ti particles. The difference between (a) Ti 5 A and (b) Ti 30 A was observed from their morphologies. Irregular round-shaped particles were synthesized mainly in Ti 5 A, while most cubic morphology particles were observed in Ti 30 A. The cubic-shaped particle was reported as the crystal structure of TiN [39,40,41]. To confirm the component of cubic particles, EDS elemental mapping was conducted during the FE-SEM observation. The EDS analysis results are shown in Figure 4. The presence of nitrogen elements on the surfaces of Ti 5 A and Ti 30 A was detected. Comparing with the Ti 5 and Ti 30 A samples, the nitrogen element represented by red dots was finely dispersed in the 30 A condition, while a relatively weak nitrogen signal was seen in the 5 A sample.

In Figure 3c,d in Al particles, the particles were formed in a spherical shape in both Al 5 A and Al 30 A samples. Figure 3e,f shows the Cu samples. Spherical copper particles and grain-like particles were observed in both current conditions. All the obtained particles shown in Figure 3 had an aggregated form and size ranging from nanometers to micrometers.

A hundred particles randomly selected in FE-SEM images were measured for their size. The fine particles obtained in the reaction were in a size range of 6~139 nm in the case of using the Ti electrode and from 10 to 79 nm in the case of using the Al electrode. The average sizes of prepared nitride nanoparticles and their standard deviations are indicated in Table 2. The size of the cubic particle was defined as the length of the longest diagonal. The average size of the obtained nitride nanoparticles was approximately several tens of nanometers.

In the micro-size particles, the shape of the individual particles aggregated was the same as that of the nano-size particles. The morphologies of individual nano-size particles observed from TEM images are described below.

#### 3.1.2. FE-TEM Observation

##### Ti 5 A and Ti 30 A

**Figure 5:**
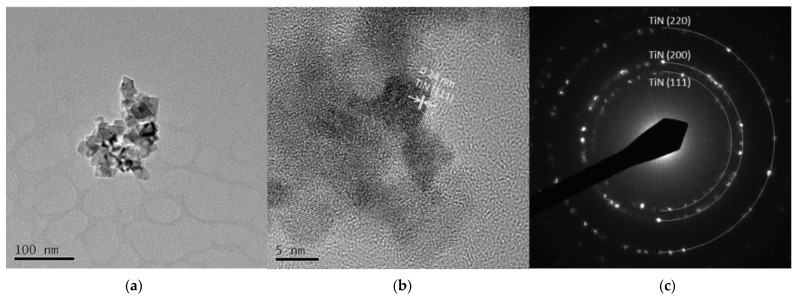
TEM images of Ti 5 A particles (**a**) low resolution image; (**b**) high resolution image; (**c**) SAED pattern.

**Figure 6:**
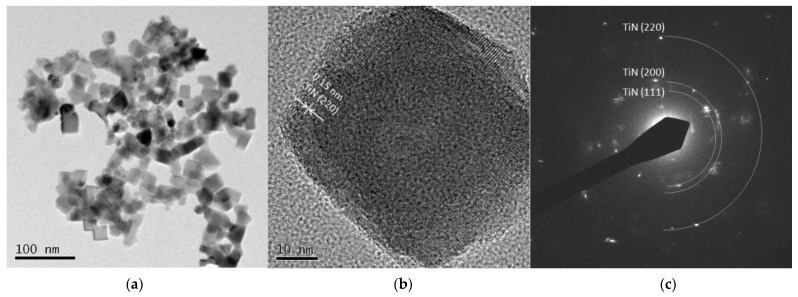
TEM images of Ti 30 A particles (**a**) low resolution image; (**b**) high resolution image; (**c**) SAED pattern.

Figure 5 and Figure 6 are TEM images of Ti 5 A and Ti 30 A, respectively. In both conditions, cubic TiN particles were confirmed. The shape of the particles corresponded to the FE-SEM observation shown in Figure 3. Ti 30 A showed relatively bigger particle sizes than Ti 5 A. Figure 5b and Figure 6b show high-resolution images of the individual cubic particles. The images show that the cubic particles had crystal lattice structures. The estimated lattice plane spacing of 0.24 and 0.15 nm corresponded to the TiN (111) plane and the TiN (220) plane, respectively. The phase composition of each product was identified by diffraction patterns obtained from TEM, as shown in Figure 5c and Figure 6c. The SAED pattern showed a dotted ring pattern, which implied that the nanoparticle had crystallinity. Thus, TiN was the main component in both Ti 5 A and Ti 30 A conditions. According to the TEM observation, nanocrystalline TiN particles were successfully produced in this method.

##### Al 5 A and Al 30 A

**Figure 7:**
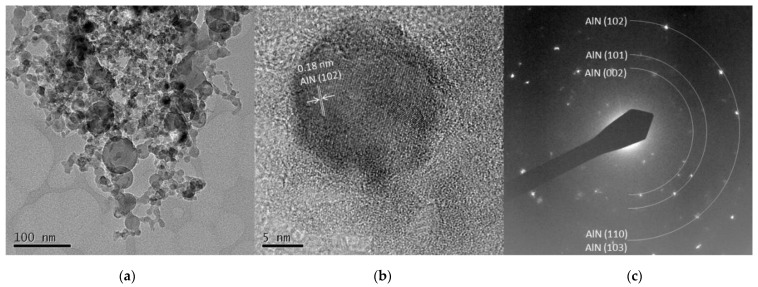
TEM images of Al 5 A particles (**a**) low resolution image; (**b**) high resolution image; (**c**) SAED pattern.

**Figure 8:**
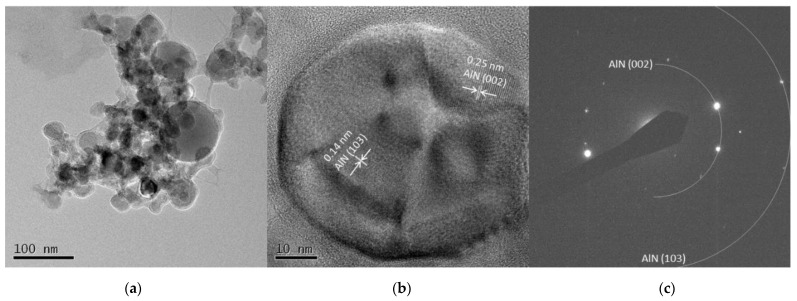
TEM images of Al 30 A particles (**a**) low resolution image; (**b**) high resolution image; (**c**) SAED pattern.

The TEM images from Al 5 A and Al 30 A are shown in Figure 7 and Figure 8, respectively. Figure 7a and Figure 8a show the synthesized spherical particles. The shape of the particles also corresponded to the FE-SEM observation shown in Figure 3. High-resolution images in Figure 7b and Figure 8b show that the spherical particles had crystal lattice structures. The estimated lattice plane spacing of 0.18, 0.14, and 0.25 nm corresponded to the AlN (102), AlN (103), and AlN (002) planes, respectively. Figure 7c and Figure 8c show the SAED pattern of a spherical particle. The diffraction patterns showed a dotted ring pattern, similar to the Ti case, inferring crystalline particles. According to the TEM observation, crystallized AlN nanoparticles were also successfully obtained.

##### Cu 5 A and Cu 30 A

**Figure 9:**
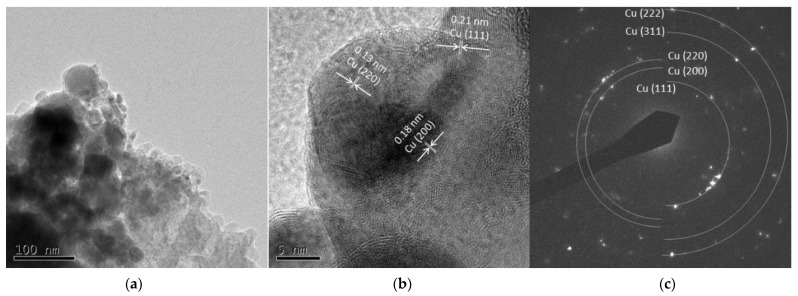
TEM images of Cu 5 A particles (**a**) low resolution image; (**b**) high resolution image; (**c**) SAED pattern.

**Figure 10:**
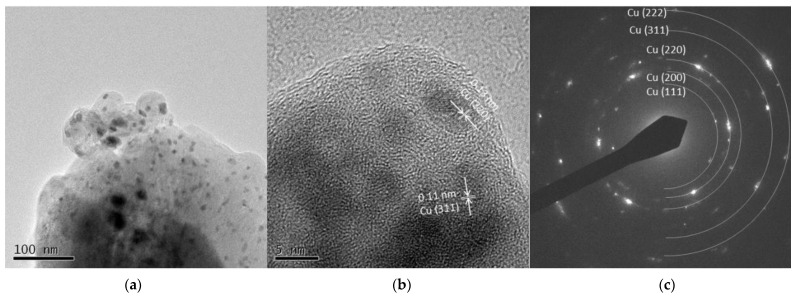
TEM images of Cu 30 A particles (**a**) low resolution image; (**b**) high resolution image; (**c**) SAED pattern.

Figure 9a and Figure 10a show spherical particles and grain-like dark particles, the latter of which were placed on the surface of the spherical particles. The shape of the particles also corresponded to the FE-SEM observation shown in Figure 3. High-resolution images in Figure 9b and Figure 10b show that the grain-like particles had crystal lattice structures. The estimated lattice plane spacing of 0.13, 0.18, 0.21, and 0.11 nm corresponded to the Cu (220), Cu (200), Cu (111), and Cu (311) planes, respectively. The SAED pattern showed a dotted ring pattern, which meant a crystalline particle. According to the TEM observation, crystallized Cu nanoparticles were obtained in this method.

### 3.2. Crystallite Phase of the Synthesized Particles in Liquid Nitrogen Discharge

#### 3.2.1. Crystallite Phase of the Synthesized TiN Particles

**Figure 11:**
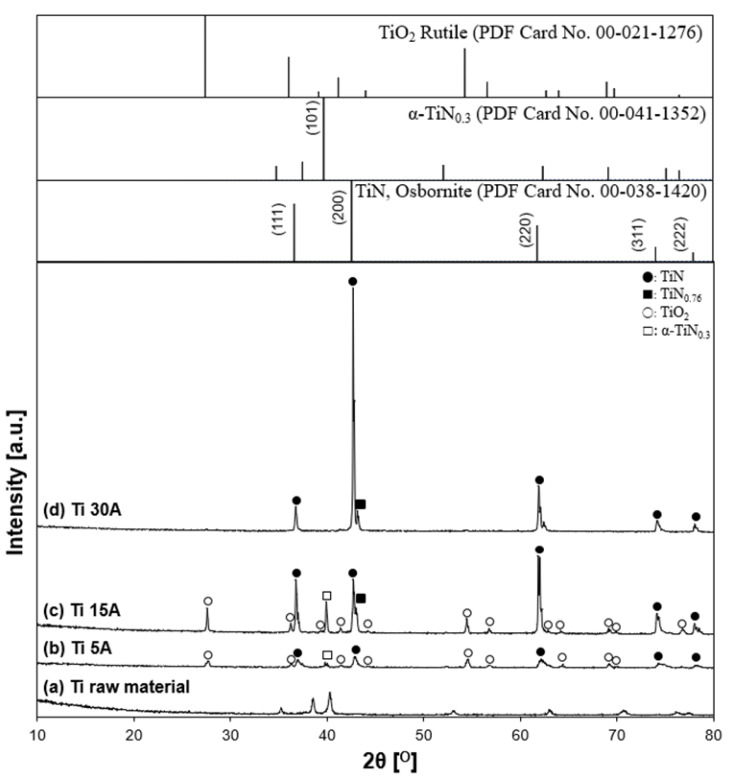
X-ray diffraction patterns of prepared samples. (**a**) Ti raw material, (**b**) Ti 5 A, (**c**) Ti 15 A, and (**d**) Ti 30 A. The inserted figure is the JCPDS PDF card of the TiN, TiN_0.76_, TiO_2_, and α-TiN_0.3_ XRD patterns. Individual crystalline phases are represented by the following characteristic peaks: (●) TiN, (■) TiN_0.76_, (○) TiO_2_, and (□) α-TiN_0.3_.

XRD characterized the coarse particle samples. Ti and Al samples at a current of 15 A were prepared to investigate the effect of the current conditions more clearly. The XRD patterns of the synthesized TiN particles and the reference patterns of nitride products are shown in Figure 11. The XRD peak of the pristine electrode surface was confirmed as an initial starting material of Ti. The presence of TiN (Osbornite, syn, JCPDS PDF card, file no. 00-038-1420, 2θ = 36.66°, 42.60°, 61.81°, 74.07°, 77.96°), which is known as osbornite, the natural structure of titanium nitride found in meteorites [47,48,49], was identified in all experimental conditions. According to the XRD patterns, Ti metallic peaks did not appear after the discharge, which indicated that Ti reacted with nitrogen and produced TiN. The crystalline phase of α-TiN_0.3_ was identified only in the 5 A and 15 A conditions. This was attributed to Ti surfaces’ partial nitriding and was considered an intermediate for the TiN nitridation process. Moreover, some Ti was oxidized to form TiO_2_ because the particles might be exposed to air during the collection. When a higher current of 30 A was applied to the electrode, the oxide peak decreased, while the nitride peak increased. The results indicated that the crystallinity of synthesized TiN was enhanced with increasing the current. Therefore, the current influenced the crystallinity of the synthesized nitride particles and the products of the TiN particles. The XRD patterns are in good agreement with the FE-SEM images in Figure 3, which showed a proportional increase in the number of cubic particles when the current was increased from 5 A to 30 A.

To confirm the observation, nitrogen contents in the Ti particles were estimated. TiN has a cubic crystal structure in the form of NaCl. Oh et al. calculated the surface energy of each plane of TiN and reported that (200) planes had the lowest surface energy compared to (111) planes in the TiN thin-film growth [50]. A similar study was conducted independently by Zhao et al. [51]. At the initial step of TiN film growth, the (200) orientation was preferred and surface energy was a dominant factor. When the film grew to a certain level, strain energy became the dominant factor; thus, the preferential facet was changed to (111) instead of (200).

According to the XRD patterns in Figure 11, increasing the current from 5 to 30 A increased the intensity of the TiN (200) peak, indicating that crystal growth proceeded preferentially on to the orientation of (200) in TiN particle formation. According to Oh’s and Zhao’s previous studies, this result implies that TiN particles synthesized by liquid nitrogen discharge are in the early stages of particle growth. It is also possible that the rapid quenching by the surrounding liquid nitrogen affects particle size control.

Wriedt et al. [52] propose the following Equation (1) to calculate the nitrogen content of TiN based on its lattice parameter, where α is the lattice parameter and x is the atomic percentage of nitrogen. The lattice parameter α can be calculated using Bragg’s Equations (2) and (3). The data in Equation (2) is given from XRD analysis, where *n* is an integer determined by the order given, λ is the wavelength of the X-ray (for Cu Kα, 1.540598 Å), d is the lattice spacing, and θ is the angle between the incident ray and the scattering planes. In Equation (3), d is given as the lattice spacing calculated in Equation (2), and *h*, *k*, and *l* are Miller indices of the corresponding plane. The information of miller indices as well as their database is given in the XRD results.

(1) ɑ = 0.4159+0.000164x

(2) nλ=2dsinθ

(3) $$d_{hkl}=\frac{a}{\sqrt{h^{2}+k^{2}+l^{2}}},$$

The calculated nitrogen content is summarized in Table 3. In the determination of the lattice constant, the single-crystal phase of TiN in the range of 2θ = 10° to 120°, (111), (200), (220), (311), (222), (400), (331), and (420), was represented. The lattice constant was measured and calculated by averaging all values from each diffraction peak of the plane. The lattice constant calculated in the 30 A condition was 4.2334 Å. This value is approximately 45 at.%, which is in good agreement with the standard reference value of 4.2417 Å (JCPDS PDF card #00-038-1420). According to the calculated data from Table 3, a higher-current condition provided higher nitrogen contents, which corresponded to the TiN crystallinity of the XRD patterns.

#### 3.2.2. Crystallite Phase of the Synthesized Al/AlN Particles

**Figure 12:**
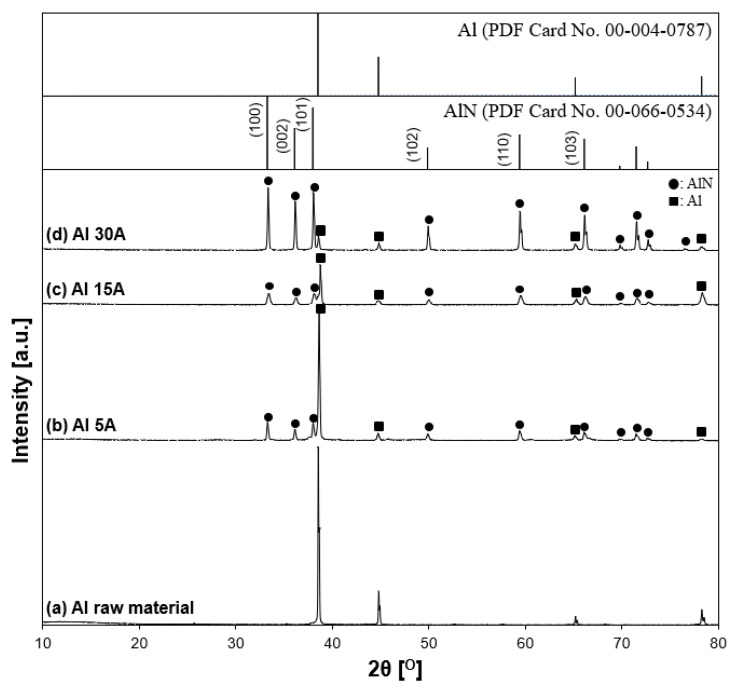
X-ray diffraction patterns of synthesized samples. (**a**) Al raw material, (**b**) Al 5 A, (**c**) Al 15 A, and (**d**) Al 30 A. The inserted figure is the JCPDS PDF card of the Al and AlN XRD patterns. The following characteristic peaks represent individual crystalline phases: (●) AlN and (■) Al.

Figure 12 shows XRD patterns of the synthesized Al/AlN particles with variable currents of 5, 15, and 30 A. According to the characterization results, Al and AlN were the main components. The peaks of the AlN (aluminum nitride JCPDS PDF card, file no. 00-066-0534, 2θ = 33.22°, 36.05°, 37.94°, 49.83°, 59.36°, 66.07°, 69.75°, 71.45°, 72.64°, 76.48°) appeared in every experimental condition. The XRD patterns showed that when the current increased, the intensities of AlN peaks increased, while those of Al peaks decreased, indicating that the crystallinity of synthesized AlN was enhanced with increasing current, similar to the Ti experiments. However, in the Al 30 A sample, unreacted Al remained.

#### 3.2.3. Crystallite Phase of the Prepared Cu Particles

**Figure 13:**
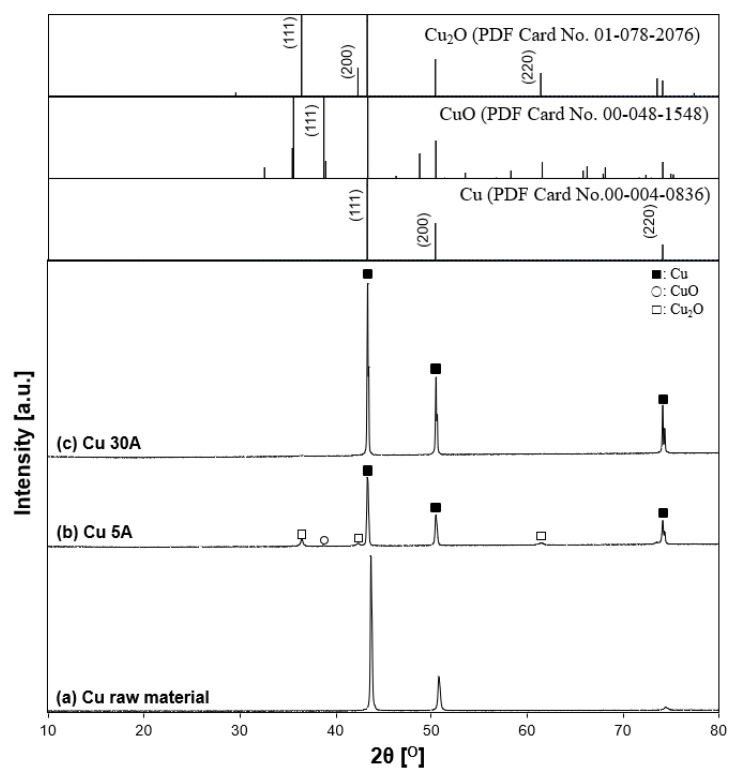
X-ray diffraction patterns of synthesized samples. (**a**) Cu raw material, (**b**) Cu 5 A, (**c**) and Cu 30 A. The inserted figure is the JCPDS PDF card of the Cu, CuO, Cu_2_O XRD patterns. Individual crystalline phases are represented by the following characteristic peaks: (■) Cu, (○) CuO, and (□) Cu_2_O.

Figure 13 indicates the XRD patterns of the prepared copper particles. According to the diffraction patterns shown in low-power mode, the particles contained the crystallite phases of Cu (copper, JCPDS PDF card no. 00-004-0836, 2θ = 43.3°, 50.4°, 74.1°), Cu_2_O, and CuO. The Cu_2_O peak showed relatively higher intensity than the CuO peak for the oxide state peaks, suggesting that the particles were partially oxidized. The reason was thought to be due to the sampling procedure in the process. Liquid nitrogen strongly moved up and down with boiling during the experiment, making the black fumes vaporize. The fume’s contents, which were thought to be fine nanoparticles, were mostly stacked on top of each other around the vessel’s edge. At the end of the experiment, the majority of the residual particles were attached in three locations: the vessel bottom, the vessel wall below the liquid nitrogen surface, and the vessel’s top edge boundary. In the case of the 5 A current, particle production was insufficient to obtain particles. Thus, the particles at the top boundary of the vessel were also collected. However, compared to the other two positions, the circumstance seemed different. The boundary region may be more susceptible to contact with moisture and air, resulting in oxidation. Metallic Cu peaks, conversely, appeared at a current at 30 A. Similar to the Ti case, as a higher current of 30 A was applied to the electrode, the intensity of the oxide peak decreased, while the intensity of the metallic Cu peak increased. The results showed that increasing the current increases the crystallinity of Cu.

### 3.3. Particle Preparation Using Different Electrode Combinations

#### 3.3.1. Morphologies of the Prepared Particles

**Figure 14:**
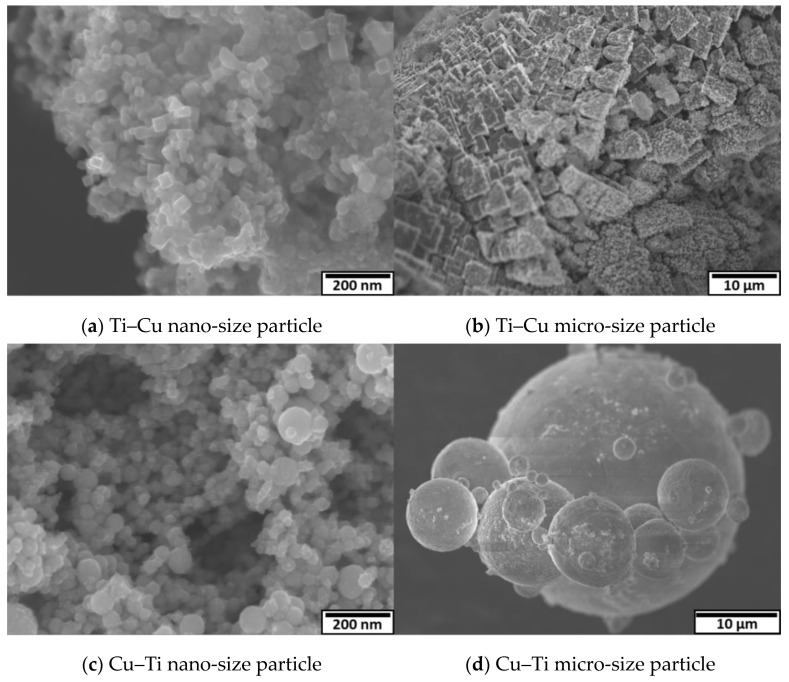
Morphologies of the prepared particles. (**a**) Ti–Cu nano-size particle, (**b**) Ti–Cu micro-size particle, (**c**) Cu–Ti nano-size particle, and (**d**) Cu–Ti micro-size particle.

Figure 14a,b shows FE-SEM (JSM-7500F) images of Ti–Cu 30 A particles from nano-size to micro-size, which were directly collected from the reactor vessel without dispersing in ethanol. At both the nano- and the micro-scale, TiN cubic particles were observed, which indicated the particles were mainly formed from the Ti anode. Most of the cubic particles were observed in agglomerated form. Figure 14c,d shows FE-SEM images of Cu–Ti 30 A particles from nano-size to micro-size particles. Cu spherical particles were mainly observed at both scales, which also inferred that the anode produced most of the particles in this process.

#### 3.3.2. Crystallite Phase of the Synthesized Particles

**Figure 15:**
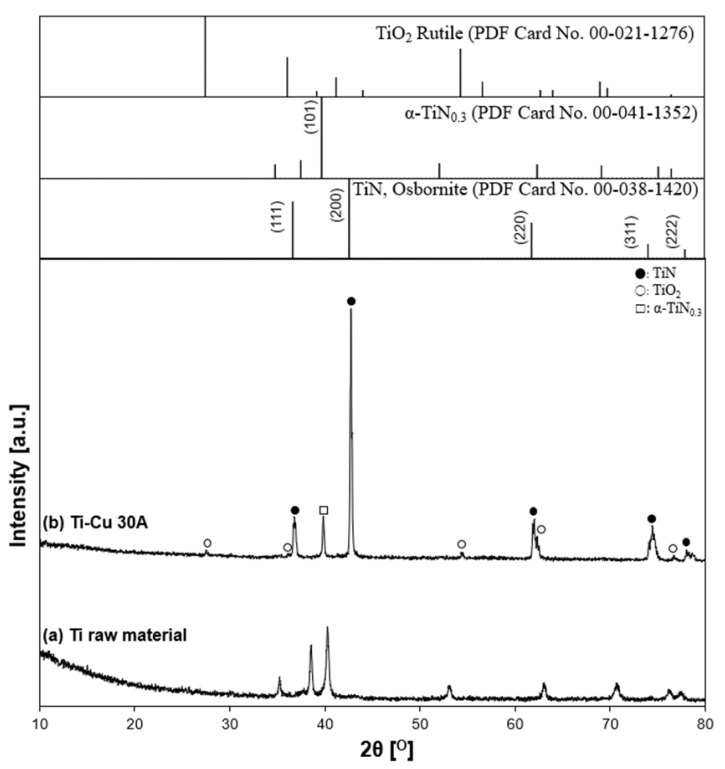
X-ray diffraction patterns of the Ti–Cu sample. (**a**) Ti electrode raw material and (**b**) Ti–Cu 30 A: (●) TiN, (○) TiO_2_, and (□) α-TiN_0.3_.

The XRD patterns of the prepared particles in the Ti–Cu 30 A condition and the initial state of the Ti anode surface, and the reference patterns of TiN, TiO_2_, and α-TiN_0.3_ are shown in Figure 15. Ti-related peaks, such as the high-intensity peaks of TiN and α-TiN_0.3_ and the low-intensity peaks of TiO_2_, were identified. However, Cu cathode-related peaks and alloys were not detected, indicating that the two different electrode materials did not involve each other in the particle generation process. The XRD pattern tendency was close to the Ti 15 A and Ti 30 A conditions, as shown in Figure 11. The calculated lattice parameter was 4.2329 Å, indicating 45% of nitrogen content by inserting the calculated lattice parameter into Equation (1). The value was almost the same as that in the previous Ti 30 A sample.

**Figure 16:**
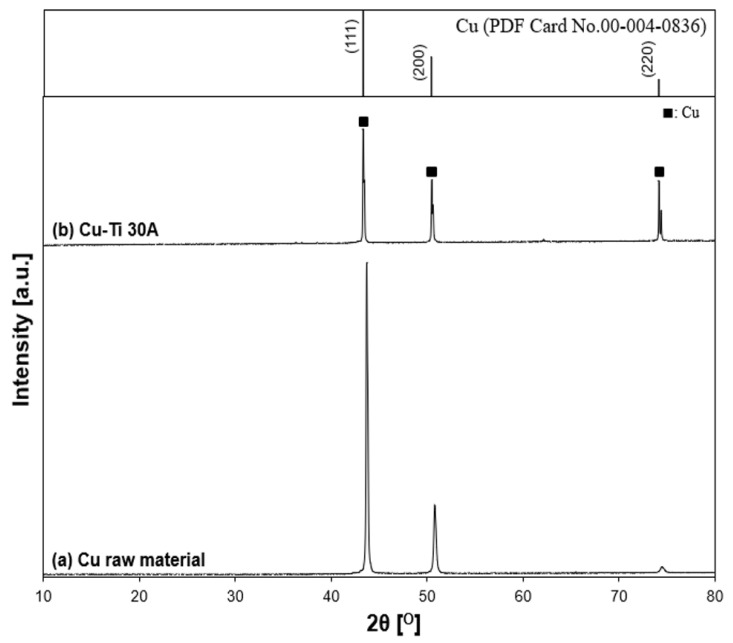
X-ray diffraction patterns of the Cu–Ti sample. (**a**) Cu electrode raw material and (**b**) Cu–Ti 30 A: (■) Cu.

The XRD patterns of prepared particles for a Cu–Ti electrode arrangement, the initial state of the Cu anode surface, and the reference patterns of Cu are shown in Figure 16. According to the XRD results, Cu was the only component of the prepared particle. There were no peaks related to the metal alloys, which was similar to the Ti–Cu electrode arrangement.

Both the results (Ti–Cu and Cu–Ti) concluded that the particles were synthesized from the anode (Ti→TiN, Cu→Cu).

## 4. Discussion

### 4.1. Mechanism of Formation Phenomena of Nitride Particles

**Figure 17:**
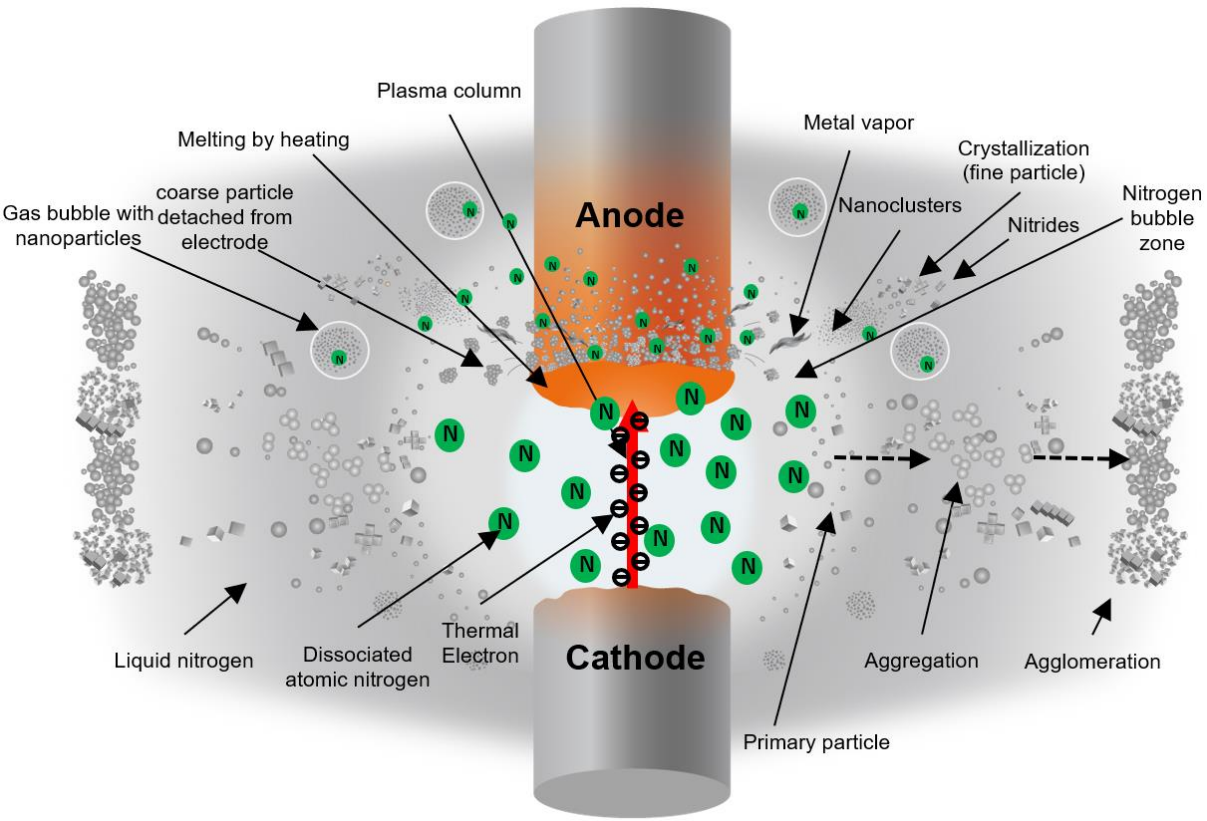
Illustration of the supposed particle formation phenomena in the submerged arc discharge process.

Based on the FE-SEM and XRD results, it is clear that the anode generates the particles in this process. Figure 17 depicts a proposed illustration of the arc discharge process in liquid nitrogen and the mechanism for metal nitride particle formation. The thermal electron (thermion) emerges from the cathode and collides with the anode surface in the arc discharge process. Particles are then produced from the heated anode surface by melting and vaporizing the anode material. The arc plasma’s temperature is thought to be high enough to melt and evaporate the arc spot at the electrode.

When the arc initiates discharge between the electrodes, a specific gas bubble area, as depicted in the white zone in Figure 17, is most likely formed between the electrodes. Inside the bubble, there is a lot of nitrogen gas. Because the arc discharge persists in the plasma column, the generated thermion can continuously dissociate nitrogen molecules into atoms as long as the arc discharge persists. Atomic nitrogen can react with Ti and Al metals to form metal nitrides. The SEM observation of the anode surface confirmed this phenomenon.

**Figure 18:**
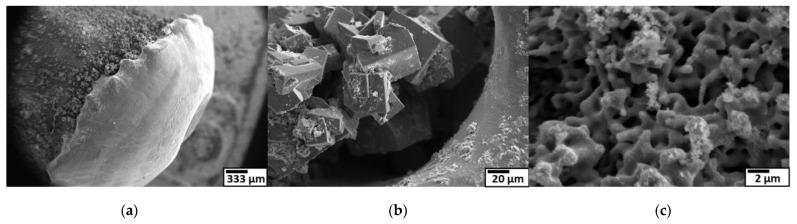
Surface morphologies of Ti anode and SEM observation at low magnification (left) to high magnification (right). (**a**) anode after discharge; (**b**) dendrite particles formed behind the melted tip; (**c**) pore structure of anode surface.

The surface of the Ti 30 A anode tip after the discharge is shown in Figure 18a. A molten trace was seen on the surface. The generated micro-sized cubic TiN particles were observed next to the melted region in Figure 18b, suggesting that the partially melted Ti reacted with nitrogen atoms and formed on the electrode surface. The generated micro-cubic particles were found in a dendrite growth structure due to the rapid cooling of gas and liquid. The size and shape of the dendrite structure particles correspond to the size and shape of the coarse particles collected. Consequently, it was thought that the coarse particles were directly derived from the detachment of those micro-sizes of cubic dendrites on the electrode during discharge, which was most likely caused by physical force such as an arcing shockwave.

Figure 18c shows that the Ti anode surface structure changed to a sponge-like pore structure, which inferred that metal vaporization occurred. Once the leached metal vapor was generated, it was rapidly quenched by the surroundings. During the supercooling process, the nucleation proceeded by condensation and formed Ti metal nanoclusters. The dissociated nitrogen atoms also reacted with generated Ti nanoclusters to form TiN. The TiN nanoclusters grew in a cubic crystal. Furthermore, produced TiN primary particles were aggregated by colliding with each other.

The particle generation mechanism by melting and vapor processes is similar for the Al and Cu electrodes. However, since AlN is decomposed at high temperature over about 2700 K, as shown in Figure 1, the particles produced using the Al electrode would be composed of both AlN and Al. Conversely, in the case of Cu, Cu did not form a nitride. Thus, the particles were obtained directly by the physical impact on the electrode without any nitride reaction involved.

### 4.2. Thermion Emission from the Cathode

**Figure 19:**
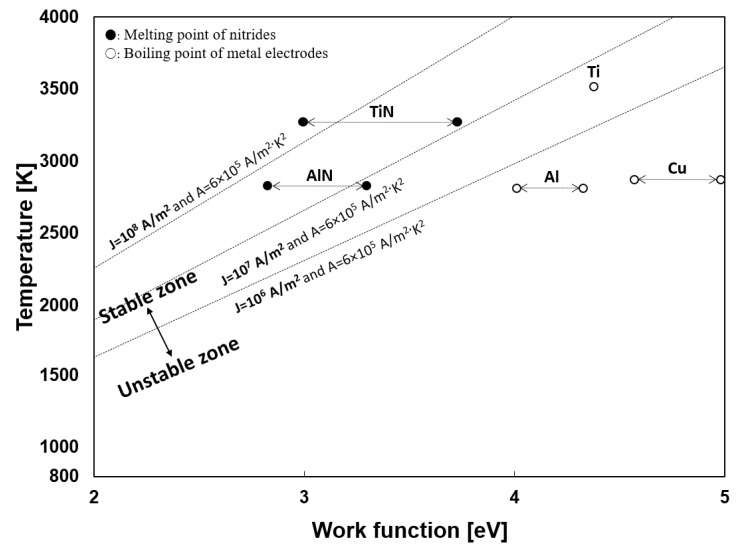
Linear function of minimum current density for stable arc discharge and the relationship between temperature and work function.

To produce the particles in the submerged arc discharge process, the thermion emission from the cathode is also essential. The stability of the arc discharge depended on the cathode materials, as described in the experimental section. Generally, the thermion emission is related to the material and temperature. Ushio et al. investigated the correlation between the arc discharge stability and cathode thermion emission [53]. Equation (4) is a Richardson–Dushman equation for the thermion emission density at the cathode [54,55,56], where *T* (K) is the cathode temperature, *W* (eV) is the work function, k_B_ (J/K) is the Boltzmann constant, and *J* [A/m^2^] is the current density at the cathode. The minimum value of the current density required for maintaining arc discharge is 10^6^ A/m^2^, as reported in many references [34,53]. The proper current density (*J*) of arc discharge is known to be 10^6^–10^8^ A/m^2^ [53]. In the equation, *A* is a thermionic emission coefficient, generally using a value of 6 × 10^5^ A/m^2^·K^2^. Figure 19 shows the relationship between temperature and the work function of the materials. The linear dot line in the middle is the minimum current density (*J* = 10^6^ − 10^8^ A/m^2^) for maintaining stable arc discharge. The work function has different values according to the plane orientation. Thus, some materials are expressed in the range, referring to other works [57,58,59,60,61].

(4) $$J=AT^{2}exp\left(-\frac{W}{k_{B}T}\right)$$

During the Ti 30 A experiment, the tip of both electrodes turned golden yellow, which is the appearance color of TiN, suggesting the tip of the Ti electrode was composed of TiN. In Figure 19, TiN requires a less work function than Ti, i.e., TiN requires less energy to emitting thermion than Ti. In other words, TiN can initiate arc discharge at a lower temperature than Ti. This was the reason why the Ti electrode provided sustainable arc discharge for the particle preparation process.

A similar discharge behavior was observed in the initial stage of the Al 30 A experiment. The white-to-pale-yellow AlN particles covered both electrodes were observed. However, as Al has a low melting point (933 K), the heated anode started melting drastically and hindered AlN formation’s effect on the cathode tip. When the melting started, the gap distance was hard to manage, causing the arc discharge to be maintained only for a few seconds.

It can be concluded that the stability of the arc discharge is affected by the cathode temperature, work function, and thermal properties of the electrode materials.

## 5. Conclusions

The submerged arc discharge process was used to synthesize nitride particles in liquid nitrogen. Changing current as an experimental parameter was used to investigate the properties of prepared particles. Using Ti and Al electrodes, TiN and AlN particles were successfully synthesized directly from metal electrodes. Particles prepared with a Cu electrode had a metallic Cu phase. When applying high-power mode, the crystallinity of the nitride peaks increased, which implied that the current influenced the crystallinity of synthesized particles. According to the experimental results of different metal electrode combinations, it was confirmed that the anode generates most of the particles in this process. Based on the results, the particle generation mechanism was proposed. The proposed particle preparation process using arc discharge in liquid nitrogen showed potential for a simple preparation process of nitride fine particles.

## Figures and Tables

**Table 1:** Detailed operating conditions.

Content	Conditions	
Dielectric medium	Liquid nitrogen	
Electrode material	Titanium rod, aluminum rod, and copper rod	
Power supply	DC	
Power mode	Low-power mode	High-power mode
Applied current [A]	5	30
Material–material (+) anode–(−) cathode	Ti–Ti Al–Al Cu–Cu	Ti–Ti Al–Al Cu–Cu Ti–Cu Cu–Ti

**Table 2:** Morphology and average particle size of prepared fine particles.

Sample	Shape	Average Size (Min.~Max.)	Standard Deviation
Ti 5 A	Sphere	30 nm (6~95 nm)	22.4
Ti 30 A	Cubic	43 nm (13~139 nm)	22.5
Al 5 A	Sphere	26 nm (10~45 nm)	6.7
Al 30 A	Sphere	46 nm (10~79 nm)	15.6

**Table 3:** Lattice parameter and nitrogen content of TiN samples.

Sample Name	Lattice Parameter (Å)	Calculated Nitrogen Content (at.%)
Ti 5 A	4.2201	37.2
Ti 15 A	4.2288	42.6
Ti 30 A	4.2334	45.3
